# Patient Selection for Downstaging of Hepatocellular Carcinoma Prior to Liver Transplantation—Adjusting the Odds?

**DOI:** 10.3389/ti.2022.10333

**Published:** 2022-04-21

**Authors:** Daniel Seehofer, Henrik Petrowsky, Stefan Schneeberger, Eric Vibert, Jens Ricke, Gonzalo Sapisochin, Jean-Charles Nault, Thomas Berg

**Affiliations:** ^1^ Department of Visceral, Transplant, Thoracic and Vascular Surgery, University Hospital, Leipzig, Germany; ^2^ Swiss HPB and Transplantation Center, Department of Surgery and Transplantation, University Hospital Zürich, Zurich, Switzerland; ^3^ Department of Visceral, Transplantation and Thoracic Surgery, Medical University of Innsbruck, Innsbruck, Austria; ^4^ Centre Hépato-Biliaire, Hôpital Paul Brousse, Villejuif, France; ^5^ Department of Radiology, LMU Munich, Munich, Germany; ^6^ Ajmera Transplant Program and HPB Surgical Oncology, Department of Surgery, Toronto General Hospital, University of Toronto, Toronto, ON, Canada; ^7^ Service d’Hépatologie, Hôpital Avicenne, Hôpitaux Universitaires Paris-Seine-Saint-Denis, Université Paris Nord, Paris, France; ^8^ INSERM UMR 1138 Functional Genomics of Solid Tumors Laboratory, Paris, France; ^9^ Division of Hepatology, Department of Medicine II, Leipzig University Medical Center, Leipzig, Germany

**Keywords:** review, liver transplantation, hepatocellular carcinoma, downstaging, transarterial chemoembolization (TACE), drop-out, intention-to-treat

## Abstract

**Background and Aims:** Morphometric features such as the Milan criteria serve as standard criteria for liver transplantation (LT) in patients with hepatocellular carcinoma (HCC). Since it has been recognized that these criteria are too restrictive and do not adequately display the tumor biology, additional selection parameters are emerging.

**Methods:** Concise review of the current literature on patient selection for downstaging and LT for HCC outside the Milan criteria.

**Results:** The major task in patients outside the Milan criteria is the need for higher granularity with patient selection, since the benefit through LT is not uniform. The recent literature clearly shows that beneath tumor size and number, additional selection parameters are useful in the process of patient selection for and during downstaging. For initial patient selection, the alpha fetoprotein (AFP) level adds additional information to the size and number of HCC nodules concerning the chance of successful downstaging and LT. This effect is quantifiable using newer selection tools like the WE (West-Eastern) downstaging criteria or the Metroticket 2.0 criteria. Also an initial PET-scan and/or tumor biopsy can be helpful, especially in the high risk group of patients outside the University of California San Francisco (UCSF) criteria. After this entry selection, the clinical course during downstaging procedures concerning the tumor and the AFP response is of paramount importance and serves as an additional final selection tool.

**Conclusion:** Selection criteria for liver transplantation in HCC patients are becoming more and more sophisticated, but are still imperfect. The implementation of molecular knowledge will hopefully support a more specific risk prediction for HCC patients in the future, but do not provide a profound basis for clinical decision-making at present.

## Introduction

Liver transplantation (LT) has become the mainstay of curative treatment for hepatocellular carcinoma (HCC) in cirrhosis, as it can provide the best long-term results (>5 years) in selected patients (1). The size and number of HCC nodules are suggestive for the risk of early tumor recurrence after LT according to the “Milan criteria” (MC) (Table 1) or the “Metroticket 2.0” criteria (2) assessments. Morphometric features have served as the main criteria for LT for many years, although from a tumor-biology viewpoint, they do not display the tumor biology. Thus, more specific selection parameters are warranted. With advances in the understanding of HCC biology, the MC appear too restrictive since a significant proportion of patients with HCC outside the MC (MC-out) are curable with LT. Recently, a prospective randomized trial confirmed that LT in selected MC-out patients markedly improved the 5-year survival from 31.2% to 77.5% (3). The major task in MC-out patients is the need for higher granularity with patient selection, since the benefit through LT is not uniform.

**TABLE 1:** Morphometric and combined (Toronto) selection criteria for LT.

	Solitary HCC	Multifocal HCC
Milan criteria (MC)	≤5 cm	Maximal 3 nodules ≤3 cm
Up-to-seven criteria (UT7)	≤7 cm	Sum of maximum tumor diameter (cm) and number of tumors ≤7
UCSF criteria	≤6.5 cm	HCC: largest nodule ≤4.5 cm and sum of the diameter of all nodules ≤8 cm
Extended Toronto criteria (eTC)	No limit in size	No limits in size and number
	Only G1 und G2 tumors (obligatory biopsy)	Only G1 und G2 tumors (obligatory biopsy)
	No tumor-associated symptoms	No tumor-associated symptoms

To overcome this issue, modifications or expansion of the MC should include parameters for estimating tumor biology and thus aid in patient selection for LT (Table 1). These parameters mainly include four categories: 1) serum biomarkers, 2) histological parameters, 3) tumor imaging, and 4) dynamic parameters during neoadjuvant measures. The following review focuses on LT candidate selection in patients initially presenting outside the MC. This includes a concise review of recently published clinical series in this field. Since many published studies are of a retrospective nature and of low quality according to the GRADE criteria (4), they do not include intention to treat (ITT) analysis and different pathways of patient selection. Hence, direct comparison of the reported results is difficult and a “meta-analysis” in the narrow sense was not considered useful for this review.

## Setting the Stage–Patho-Molecular Classification of Hepatocellular Carcinoma

Although direct investigation of tumor tissue is potentially the gold standard for HCC characterization, the investigation of biopsy material pre-LT has limitations. From the molecular and pathological characteristics, HCC is a heterogeneous tumor, both regarding the intra- and inter-tumor variability, and a major reason for the complexity of classifications; no hat fits all. Tumor development is a multistep process with malignant transformation of precursor lesions into early HCC, as described elsewhere in detail (5). During carcinogenesis and tumor progression various signaling pathways are frequently affected by recurrent somatic mutations. Despite the presence of around 50 proteins altering somatic mutations per tumor across all stages, only a few of these mutations are considered to be relevant drivers of carcinogenesis (two to six per tumor) (6). These mainly include genetic alterations in the following signaling pathways: 1) telomere maintenance, 2) Wnt/b-catenin, 3) P53/cell cycle regulation, 4) AKT/mTOR, 5) MAP kinase, 6) epigenetic modification, and 7) oxidative stress (5). Based on transcriptomic profiling, HCC subclassification interlinks dysregulation of signaling pathways with genetic alterations, histological subtypes, and prognosis underlying the molecular heterogeneity of HCC. This classification includes two major types, a proliferation and a non-proliferation type, with each encompassing different biological subclasses (Table 2). Despite these tremendous advances, biopsy-derived parameters are still underused in clinical pathways of LT and the use of genetic screening could hold important prognostic value.

**TABLE 2:** Molecular subclassification of HCC.

	“Proliferation class” (50%of HCC)	“Non-proliferation class” (50%of HCC)
G1-G6 classification	G1-G3	G4-G6
Histological/clinical characteristics	Poor differentiation	Well/moderate differentiation
	High frequency of vascular invasion	Low frequency of vascular invasion
	High AFP levels	Low AFP levels
	Frequent HBV etiology (G1-G2)	Mainly HCV and alcohol
	G3: Macrotrabecular-massive histological subtypes (poor prognosis)	G4: Contain the steatohepatitic subtypes of HCC, inflammatory infiltrates, and CRP expression
Molecular features	Chromosomal instability and TP53 mutations	Chromosomal stability
	G1: Stem cell features, RPS6KA3 mutations	G4: Retain hepatocyte-like features, IL-6/JAK/STAT activation, and rare CTNNB1 and TP53 mutations
	G1-G2: AXIN1 mutations	G5 and G6: Wnt/b-catenin pathway activation due to CTNNB1 mutations
	G3: Dysregulation of cell cycle genes, FGF19 amplification, and TSC1/2 mutations	

One reason for the ongoing lack of specific histopathological parameters prior to LT might be the intratumor heterogeneity with trunk mutations present in all cells and other private mutations present in only parts of the tumor. This results in different grades of differentiation, even within the same lesion and leads to primary or secondary resistance to systemic treatments. However, it has been repeatedly shown that even basic tumor characteristics, like poor tumor grading (G3 = poorly differentiated HCC) (7) or aneuploidy (8) are important predictors of tumor recurrence. To note, HCC analyzed from liver explants gave a good prognostic tumor score according to the molecular prognosticator five-gene score and in HCC from G4 molecular subgroups, which included small well-differentiated HCC without microvascular invasion (mVI) developed on cirrhosis and expressing a transcriptomic program close to mature hepatocytes (9). However, these results should be read with caution since some of the most aggressive tumors may not be included due to drop out from the waiting list. Using whole-genome sequencing, a recent analysis has shown that in multifocal tumors, only 20%–40% are intrahepatic metastases arising from the same clone, whereas the remaining are based on *de novo* independent carcinogenesis of the diseased liver parenchyma at different sites (10). Therefore, the predictive potential of confined biopsy material, e.g., prior to LT, has natural limits where multifocal disease is frequently observed. In patients of (known) tumor heterogeneity, the worst grade determines the prognosis (11).

**FIGURE 1:**
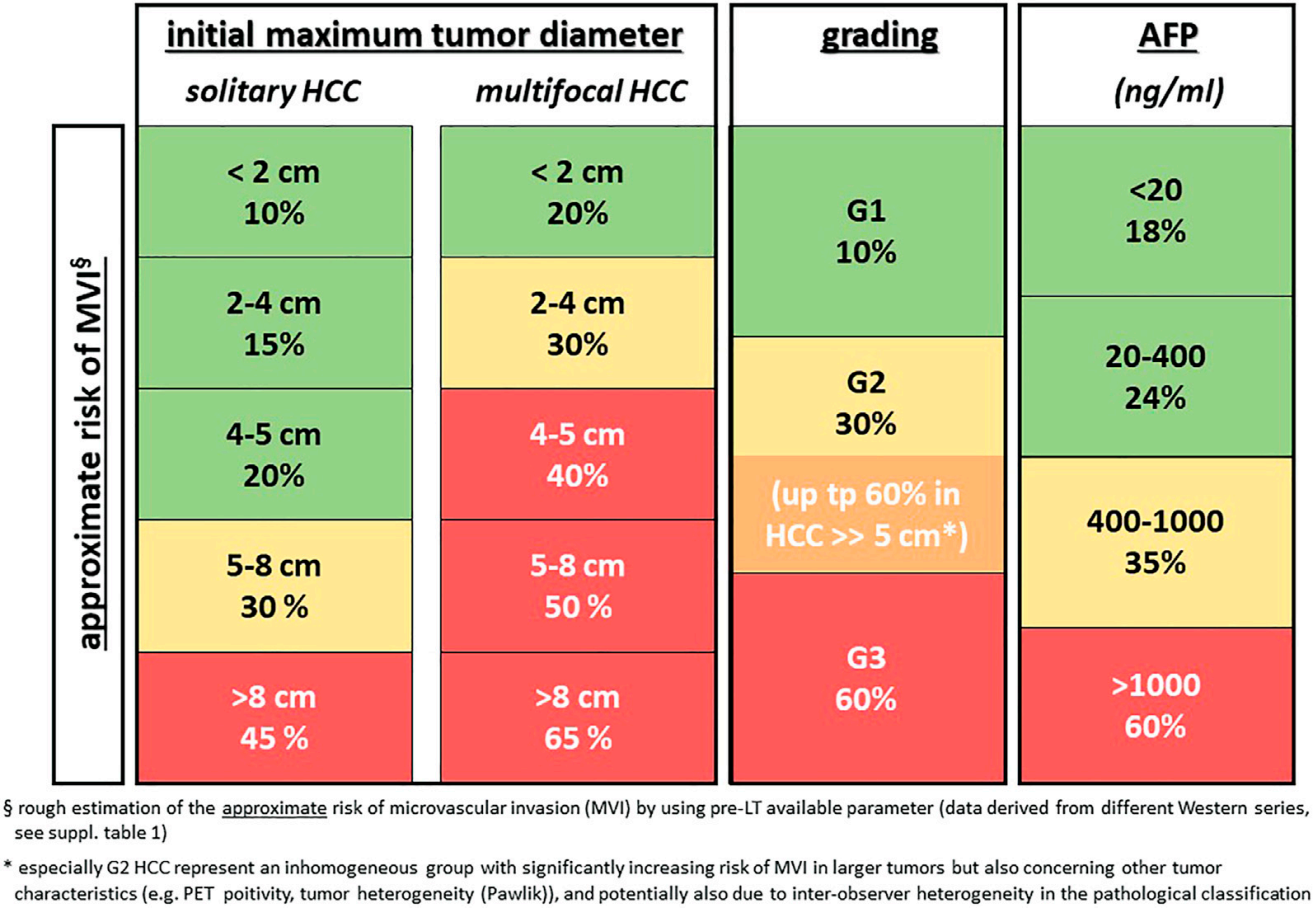
Rough estimation of the risk of microvascular invasion (mVI) by using pre-LT available parameters (data derived from different Western series, see Supplementary Table S1). * especially G2 HCC represents an inhomogeneous group with significantly increasing risk of mVI in larger tumors but also concerning other tumor characteristics (e.g., PET positivity, tumor heterogeneity [Pawlik]), and potentially also due to inter-observer heterogeneity in the pathological classification.

mVI, as one of the most relevant risk factors for HCC recurrence after LT, has been shown to be more predictive of tumor recurrence than, for example, standard staging criteria (12). While macrovascular invasion as a contraindication for LT can nowadays be diagnosed more precisely due to better imaging modalities (13), mVI can only be detected in surgical specimens and not by imaging or in biopsy material. Surrogate parameters like tumor size and number or serum alpha fetoprotein (AFP) levels are still in use for predicting the risk of mVI (Figure 1). Moreover, some new markers, such as serum and tissue PIVKA-II expression (14), combination of microRNA expression in HCC (15), or a 35-gene molecular tumor signature (16) have been proposed to predict mVI more precisely but these results still require external prospective validation. Overall, there is clearly a critical unmet need for reliable invasive or non-invasive preoperative detection of mVI and/or tumor biology taking into account biological diversity and intra- and inter-tumor heterogeneity.

## Still Not Outdated: Morphometric Parameters—Should There be an Upper Limit of Size and Number for Downstaging?

Since HCC size and number are easily accessible information by imaging, these parameters are traditionally used as a basis for further discussion and decision-making in many HCC-LT patient selection algorithms. As the risk for mVI and/or a G3 tumor corresponds with tumor size and number in unselected cohorts, these parameters ensure a relatively low drop-out rate during listing (17). While the morphometric selection criteria fulfill the idea of a great outcome per an ITT perspective, they are too unspecific as a (sole) surrogate for tumor biology. This shows the risk of withholding a life-saving procedure from a group of patients with large/multilocular but biologically favorable HCCs.

**FIGURE 2:**
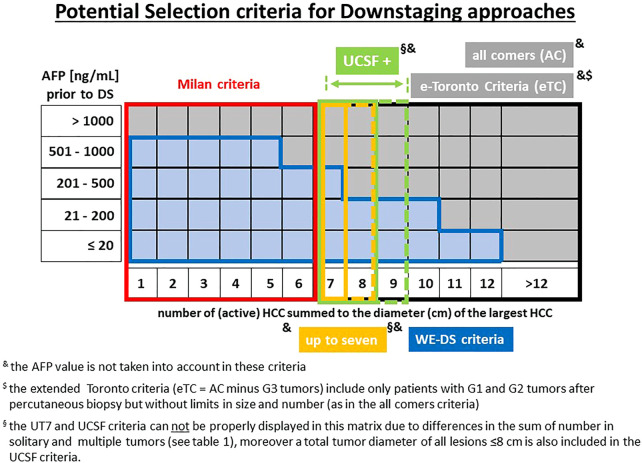
Potential criteria for downstaging-approaches prior to LT. and the AFP value is not taken into account in these criteria. $ the extended Toronto criteria include only patients with G1 and G2 tumors after percutaneous biopsy but without limits in size and number (eTC = AC minus G3 tumors). § the UT7 and UCSF criteria cannot be properly displayed in this matrix due to differences in the sum of number in solitary and multiple tumors (see Table 1), moreover a total tumor diameter of all lesions ≤8 cm is also included in the UCSF criteria.

In patients outside the MC, a majority of Western transplant centers use some form of downstaging technique before LT. Center policies for including patients in downstaging protocols vary widely (Figure 2), resulting in diverse drop-out rates. In this context, it is important to emphasize that an “acceptable drop-out rate” remains ill-defined and may be difficult to specify from an ethical perspective. The justification for downstaging and an acceptable drop-out rate needs to be seen and determined in light of the efficacy of alternative treatment methods, organ availability, and a patient’s attitude toward a concept that holds a limited chance of success. Patients outside the University of California San Francisco criteria (UCSF-out) have a significantly lower rate of successful downstaging, as described by Sinha et al. who reported that the success of downstaging decreases with increasing tumor burden. The proportion of successful downstaging after 12 months was 68% in patients with a sum of maximum diameter and tumor number ≤8 compared to 47% in patients with a sum of 12, and 38% in patients with a sum of 14 tumors (18). However, no patient selection was performed in any of these trials at the entry of downstaging (i.e., true all-comers population).

**FIGURE 3:**
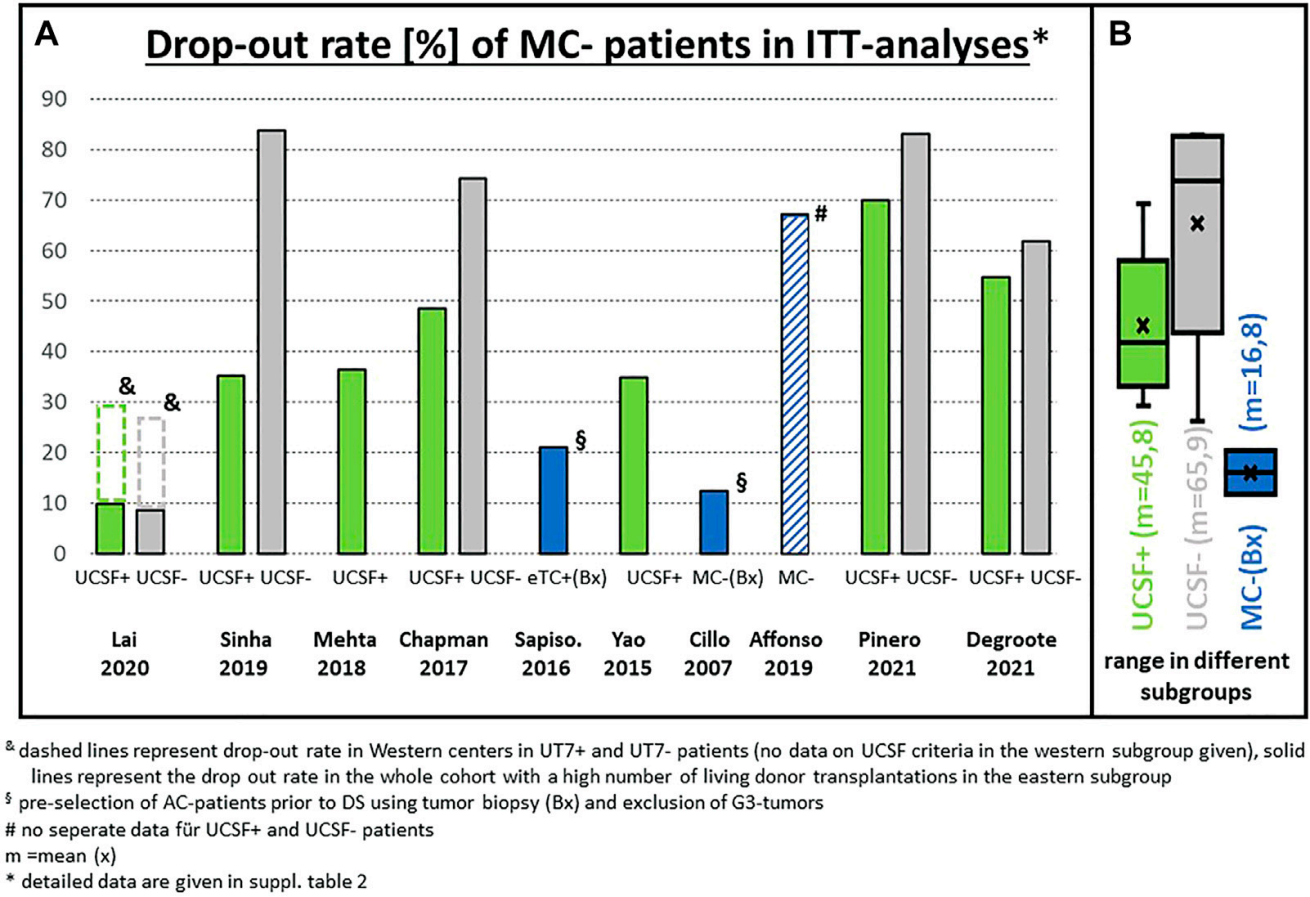
**(A)** Drop-out rate of patients outside the Milan criteria (MC) in the literature (only ITT analyses included): Percentage of patients not transplanted mainly because downstaging failed to reach the Milan criteria; **(B)** statistical summary of the different studies (box plot). & dashed lines represent drop-out rate in Western centers in UT7+ and UT7-patients (no data on UCSF criteria in the Western subgroup given). Solid lines represent the drop-out rate in the whole cohort for UCSF criteria with a high number of living donor transplantations in the Eastern subgroup. § pre-selection of AC patients prior to downstaging using tumor biopsy (Bx) and exclusion of G3 tumors. # no separate data for UCSF+ and UCSF- patients. $ the low drop-out rate of 27% in UT7-patients was observed only in one multicenter analysis (Lai, 2020) with a very short median waiting time of approximately 4 months * detailed data are given in Supplementary Table S2.

The rationale for considering the tumor burden also relates to the reported drop-out rates of 54% and 77% after 1 and 2 years, respectively for UCSF-out patients compared to 25% and 35% in UCSF-in patients (18). The overall drop-out risk in completely unselected all-comers was 84% (62 out of 74 UCSF-out patients), which can be considered unreasonably high (Figure 3). Others have described a drop-out rate of 50% or more in unselected (no biopsy, no PET, no AFP limit) patients after entering a downstaging protocol (19). The drop-out risk clearly correlates with the tumor burden on presentation.

The UCSF criteria might be considered a reasonable upper limit for applying downstaging protocols on a solely morphometric basis. However, in the all-comers cohort, a proportion of patients can be cured by LT, although the likelihood is clearly lower (20,21). Considering this, it might be ‘too restrictive’ or more precisely ‘too unspecific’, if patient selection for downstaging is only based on tumor size and number. Moreover, the dichotomous nature of such criteria, which might also be subject to indistinctness in measurement technique, does not reflect the complex tumor biology of HCC. Other and more specific parameters to modulate the entry risk are therefore required, but relevant clinical data are only available for tumor grading, AFP levels, and PET-CT (see below).

## Implications for Tumor Biopsy Before Initiation of Downstaging

**FIGURE 4:**
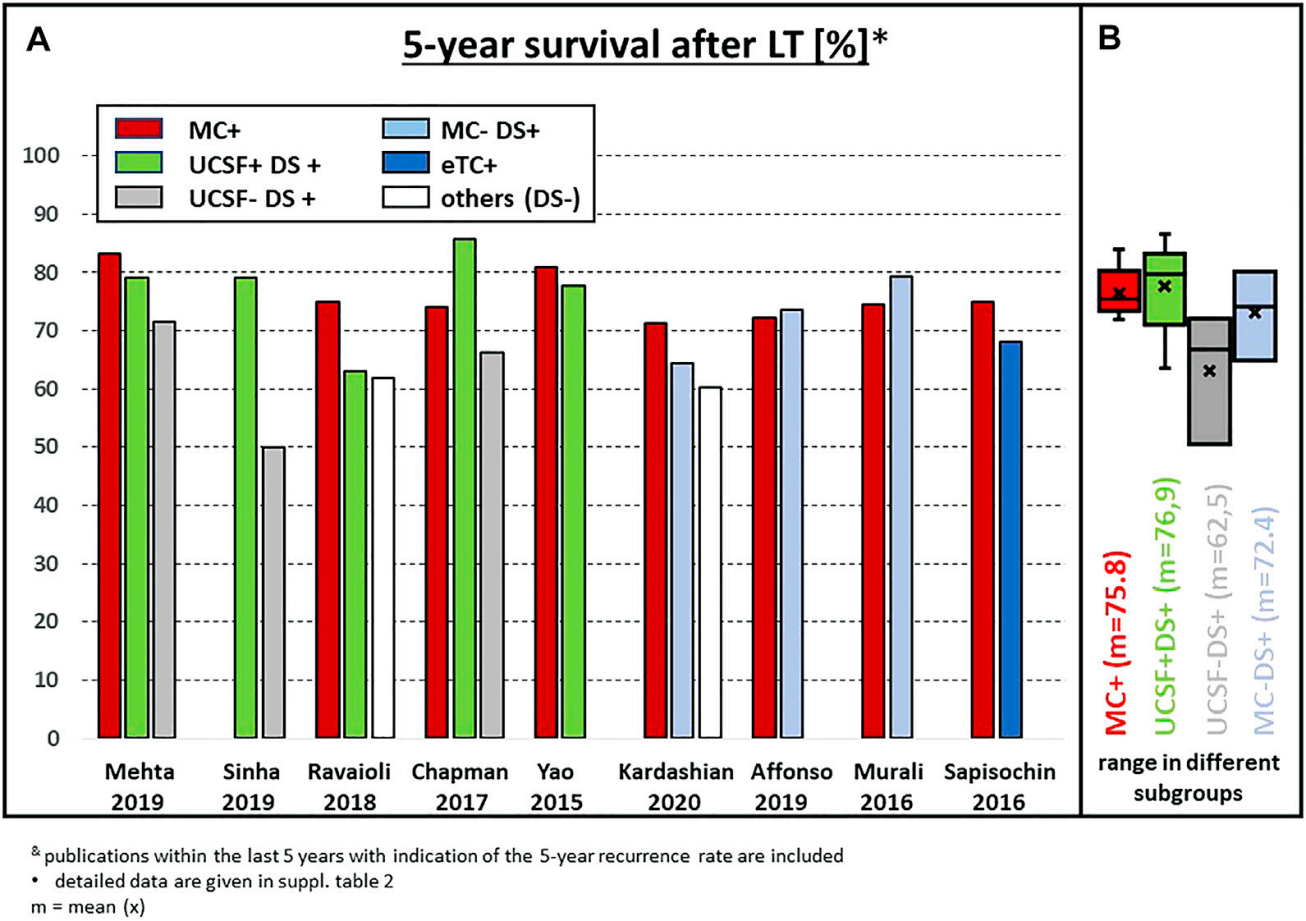
**(A)** Actuarial 5-year survival after LT in recently published series of patients with subgroups outside the MC; **(B)** statistical summary of the different studies (box plot). & publications within the last 5 years with indication of the 5-year recurrence rate are included. * detailed data are given in Supplementary Table S2.

**FIGURE 5:**
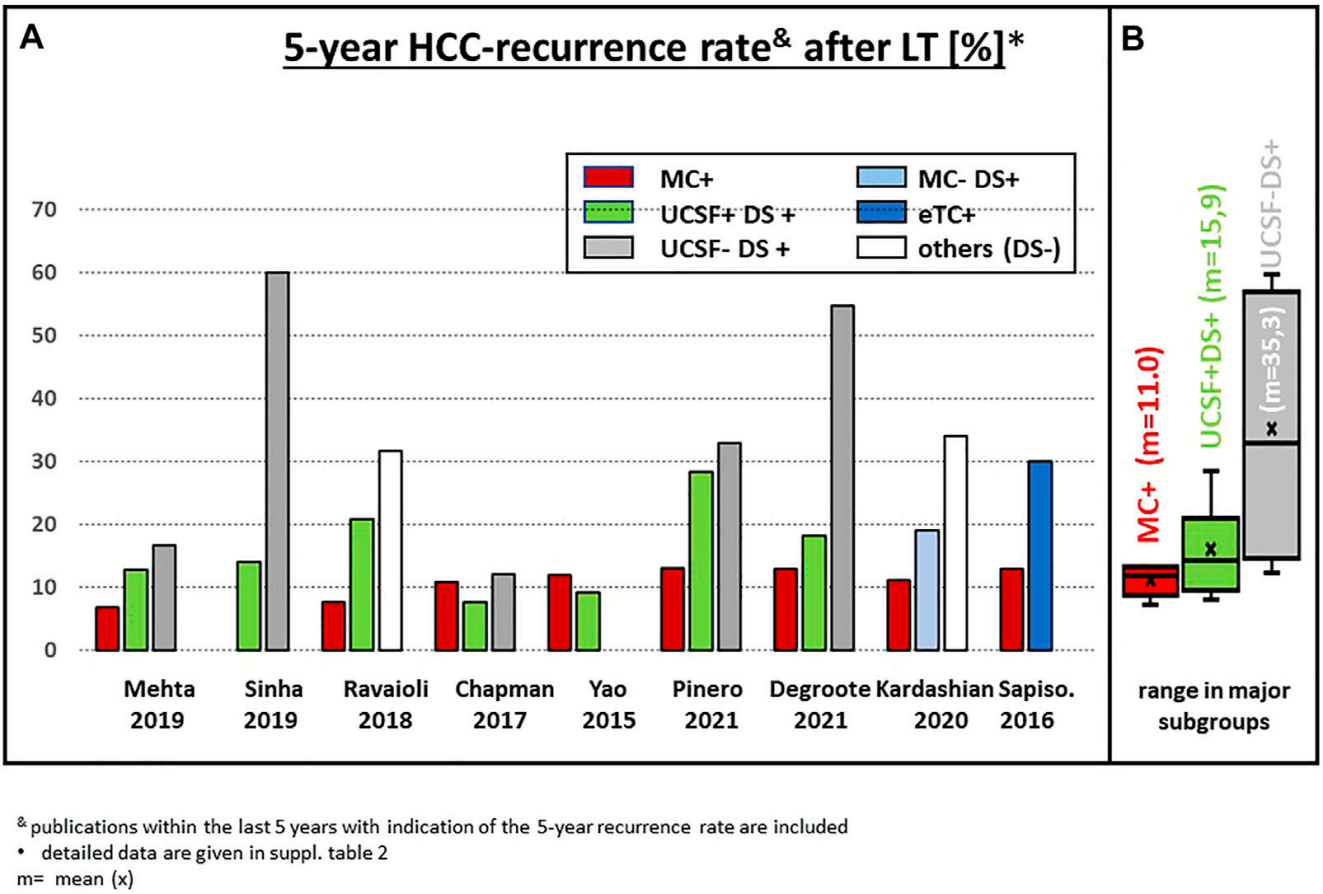
**(A)** Actuarial 5-year HCC recurrence rate after LT in recently published series of patients with subgroups outside the MC; **(B)** statistical summary of the different studies (box plot). & publications within the last 5 years with indication of the 5-year recurrence rate are included. * detailed data are given in Supplementary Table S2.

Considering the available data, the only biopsy-generated parameter with sufficient evidence for patient selection in LT is (poor) tumor differentiation. However, despite the progress in the patho-molecular classification of HCC, the exclusion of macrotrabecular-massive subtypes as a well-defined histological entity (22), which can be assessed on biopsy, should be deliberated. This subtype is associated with a poor prognosis after resection and ablation (23), but until now this has not been tested in a pre-transplantation setting. Since HCC is one of the rare tumors, where the final diagnosis can be made on the basis of non-invasive imaging, many centers do not perform a tumor biopsy prior to LT. This is, among others, based on the potential hazard of tumor cell dissemination. The risk of tumor seeding is undeniable, and cases of needle tract metastases have been described. However, the risk of (isolated) needle tract seeding and HCC recurrence after LT in two large cohorts was only 1.3% (24) and 1.9% (7) when using an adequate biopsy technique. Patients within the MC might not need a tumor biopsy, due to the relatively low risk of G3 tumors, and the acceptable risk of mVI in those with small G3 tumors. In some studies, these patients have also shown that with relevant waiting time, the test of time or the dynamic response to bridging therapy ensures adequate patient selection, proven by the excellent long-term outcome without pre-LT biopsy (Figures 4, 5). However, in patients within the MC, the very low risk has to be weighed against the potential benefit of tumor biopsy, with the seeding risk being negligible in MC-out patients.

Two single center experiences showed that upfront exclusion of G3 tumors in MC-out patients as an entry criterion for downstaging protocols might be a key factor to improve the survival results. Indeed, using this approach, Cillo et al. reported a 75% 5-year survival after LT irrespective of size and number (25). More recently, the updated Toronto experience confirmed these data, with 5- and 10-year actuarial patient survival rates of 68% and 50%, respectively in the MC-out group, which was slightly but not significantly different from the MC-in group (76% and 60%) (7) (Figure 4). However, pretransplant biopsy results might also be misleading due to tumor heterogeneity (26) and thereby produce a relevant number of false-positive or false-negative results. In the experience of Court et al., only 29% of G3 tumors were diagnosed correctly by pre-LT needle biopsy, while 17 out of 155 (11%) tumors with G1 or G2 differentiation in the final explant histology were classified as G3 by needle biopsy (27). In addition, the recurrence rate in this analysis did not correlate with the pre-LT biopsy, but with the grading in the final explant pathology.

Nevertheless, two prospective analyses have shown that a biopsy and eventually repeated biopsies are able to exclude a substantial proportion but not all G3 tumors. In the Toronto [7] and Padua cohorts (25), only 8% and 16% of MC-out patients were finally found to have a G3 tumor, respectively, even though the initial pre-LT biopsy did not show a G3 tumor. Not surprisingly, the few patients with G3 tumors had a 5-year disease-free survival of 47% compared to 82% in non-G3 patients (*p* = 0.008) (7). In comparison, the multicentric analysis by Mazzaferro et al. revealed a 27% incidence of G3 tumors in the MC-out subgroup (28). None of the available biopsy-driven series capture the “true ITT-population” including those patients with G3 tumors on biopsy, which were excluded from further downstaging. Therefore, this quota remains elusive.

But, conversely, a preoperative biopsy was 84%–92% effective in excluding G3 lesions. In addition, the incidence of mVI was only 26% in the MC-out patients and not statistically different from the MC-in group (25). Besides the excellent long-term results, the drop-out rate on an ITT basis was relatively low in both series with 21.4% and 12.5% in MC-out patients, indicating an effective reduction of the entry risk prior to downstaging (Figure 3). In contrast, series without any selection prior to downstaging revealed drop-out rates of more than 50% in the UCSF-out subgroup (Figure 3), relating to a drop-out reduction risk of approximately 50%–60% from the initial risk. The Padua cohort also showed that the risk of drop out increases with waiting time, leading to a 12.5% drop-out rate at 18 months and a 40% drop-out rate at 24 months (25). However, the 3- and 5-year survival rates on an ITT basis were not significantly different between the UCSF-out (85% and 76%) and the UCSF-in (85 and 85%) groups, suggesting that in pre-selected cohorts, e.g., by exclusion of G3 tumors, the UCSF criteria do not seem to be an ideal discriminator.

In summary, excluding G3 tumors using tumor biopsies means the entry risk in all-comers populations can be reduced to a risk comparable to UCSF-in patients in terms of drop out. In addition, the percentage of mVI and (overlooked) G3 tumors might be reduced to a level comparable to MC-in patients. From the present data, exclusion of G3 tumors might be beneficial in UCSF-in patients, and particularly useful in UCSF-out patients prior to downstaging. However, controversy remains as to whether tumor biopsy is the ideal method, or if non-invasive parameters might be comparably adequate.

## Can the Role of the Initial AFP Level Act as a Gatekeeper?

High AFP levels are known to be associated with tumor aggressiveness, poorly differentiated tumors, and mVI. Accordingly, it has been clearly shown that AFP provides prognostic information beyond tumor size and number (29). The prognostic value of an increasing AFP during waitlisting but also the AFP level at the time of LT has been shown in several analyses (see below). The establishment of the Hazard Associated with Liver Transplantation for HCC (HALT-HCC) score suggests that the addition of AFP levels facilitates the identification of patients with a poor prognosis within the MC and also of patients with a favorable prognosis outside the MC, using a cut-off HALT score of 17 (30). Consequently, AFP levels at LT have gained relevance for organ allocation. However, data on the initial AFP level and its relevance on patient selection for downstaging are less clear. Analyses of patients with the majority of patients undergoing living donor liver transplantation mainly without bridging or downstaging therapy also move towards inclusion of biological parameters, mainly the AFP value, e.g., in the Japanese “5-5-500 rule” (31). The impact of waiting time (i.e., living vs. deceased donor LT) on tumor recurrence outside the MC is also an interesting issue, but clearly beyond the scope of the present review.

On the one hand, AFP might help to identify patients with a high drop-out risk during downstaging, and on the other hand, an initial AFP <20 ng/ml might be predictive of a good response to downstaging therapies and a low recurrence rate after LT. About one-third of HCC patients inside or outside the MC present with normal AFP levels (<20 ng/ml) (32). It has been shown that in AFP-negative tumors the proportion of G3 tumors is significantly lower than in AFP-positive tumors (15% vs. 28%, *p* < 0.001). Accordingly, the rate of vascular invasion was significantly lower (20% vs. 31%, *p* < 0.001) and the percentage of pathological complete tumor necrosis was significantly higher (25 % vs. 16%, *p* = 0.01) in AFP-negative patients [32]. In a French cohort, when AFP levels were <100 ng/ml, only 2% of patients had a G3 tumor and only 20% of patients had mVI (plus 5% macroinvasion) (33).

Despite this, there still seems to be a subgroup of AFP-negative patients with impaired prognosis after LT. Additional initial selection criteria like AFP-DCP/PIVKA-II (34), PET-CT, or tumor biopsy might be of help with this specific subgroup but has yet to be fully investigated. In contrast, the response to downstaging as a predictive factor was also confirmed in AFP-negative tumors, as a tumor burden outside the MC at LT is a major risk factor for HCC recurrence (HR 10, 0.0; 3.7–33.3; *p* < 0.001). In the whole AFP-negative group, no recurrence was observed in the subgroup of patients with negative AFP and successful downstaging (32). Data generated from a US multicenter analysis unraveled that an AFP <20 ng/ml was a predictor of complete pathologic response (cPR) [63]. The rate of cPR was 26.6% in AFP-negative tumors, but only 19.5% in tumors with AFP >20 ng/ml at any time, but the majority of patients were MC-in. However, the percentage of AFP-negative tumors seems to be around 30% in MC-out and MC-in patients (32).

In AFP-producing tumors, it is increasingly clear that the predictive value of morphometrical parameters can be refined by simultaneous consideration of the AFP level. Likewise, an AFP level of >1,000 ng/ml at the time of LT has been validated as a poor prognostic factor in MC-out patients (33), as well as in MC-in patients, even though the incidence of AFP >1,000 ng/ml in MC-in patients was below 5% (29). This cut-off of 1,000 ng/ml or an increasing AFP prior to LT is generally accepted as a poor prognostic factor. In MC-out patients, the risk of tumor recurrence gradually increases with AFP values between 20 and 1,000 ng/ml at LT, thereby providing a risk stratification within defined criteria of tumor size and number. The additional value of the AFP level to improve patient selection in this context was first shown in the French AFP model, in which the upper limit of size and number could be increased from the MC criteria to ≤6 cm in cases of ≤3 nodules and to ≤3 cm in cases of ≥4 tumor nodules in patients with pre-LT AFP levels ≤100 ng/ml without a significantly increased risk of HCC recurrence (33). However, validating studies revealed a poor predictive value of this model, also pointing out the importance of the underlying liver disease (e.g., HCV vs. non-HCV) in different cohorts (35). In view of the fact that HCV is also displaced by NASH in the LT population (36), this might become extremely relevant, since many of the models are derived from cohorts with high numbers of patients with viral hepatitis. Other series have focused on an AFP limit of <400 ng/ml and found a low 5-year recurrence rate of 4.9% in patients with a total tumor diameter of <8 cm (37), or a 4-year recurrence rate of 9.4% in patients with a total tumor volume (TTV) of <115 cm^3^ and AFP <400 ng/ml (38).

**FIGURE 6:**
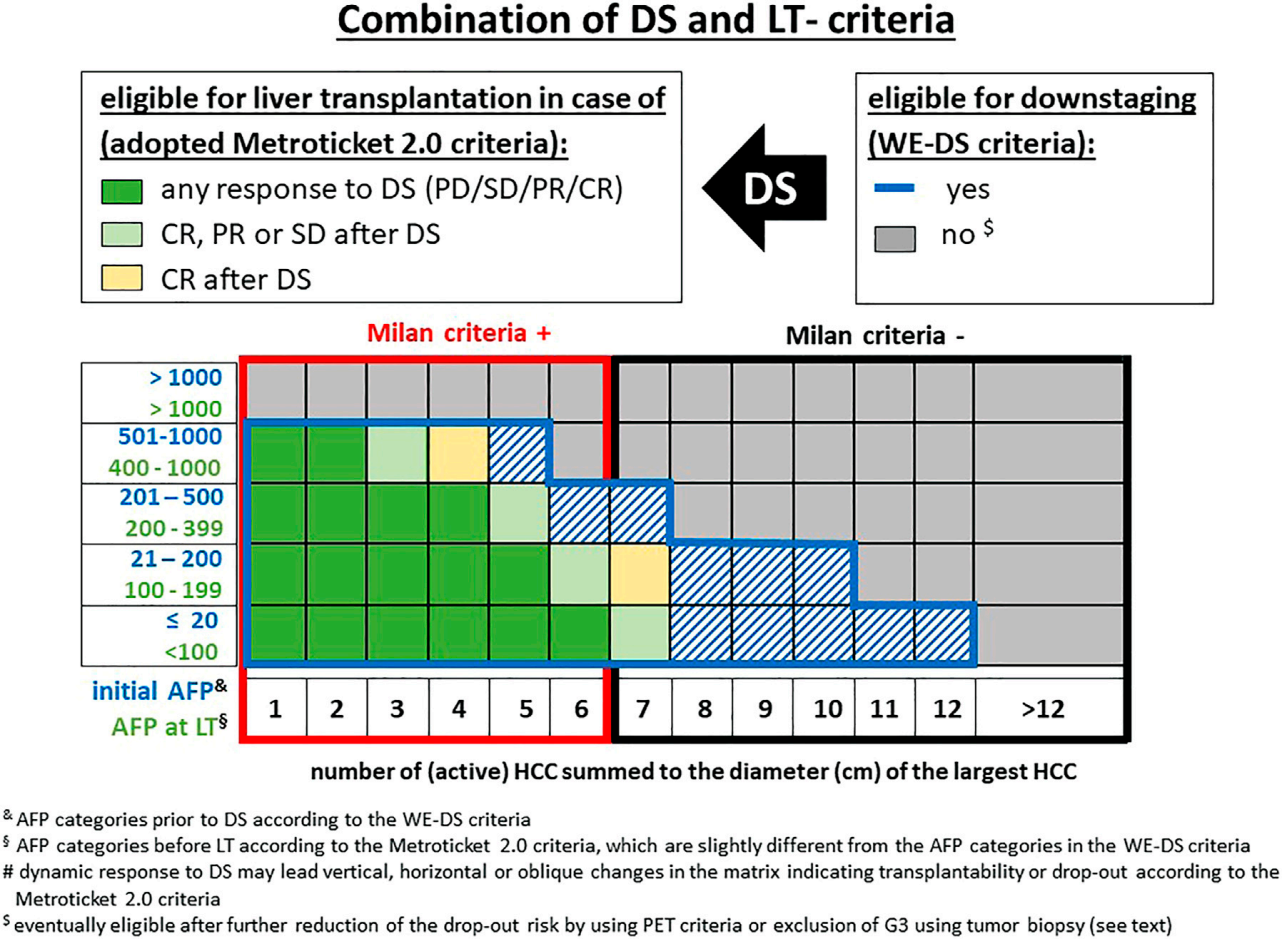
Combination of published downstaging and LT criteria (DS: downstaging, PD: progressive disease, PR: partial response, CR: complete response). & AFP categories prior to DS according to the WE-DS criteria. § AFP categories before LT according to the Metroticket 2.0 criteria, which are slightly different from the AFP categories in the WE-DS criteria. $ eventually eligible after further reduction of the drop-out risk by using PET criteria or exclusion of G3 using tumor biopsy (see text).

Lai et al. (39) raised the point that size and number alone are insufficient selection parameters and the AFP levels at first referral might overcome or at least reduce this problem. In a multicentric analysis of 3091 HCC patients at 12 centers, an ITT model was used for an upper limit of tumor burden for downstaging. A successful LT was defined as a 30% 5-year survival after LT and recalibrated to >13% 5-year survival rate after the time of first referral, otherwise LT was estimated to become an unrealistic goal. In this model, the upper limit of tumor burden at presentation revealed an inverse relation with the initial AFP level. Whereas in patients with an AFP level ≤20 ng/ml, an up to 12 sum of HCC number and diameter was acceptable, which decreased with increasing AFP to 10 (AFP 21–200 ng/ml), 7 (AFP 201–500 ng/ml), and 5 (AFP 501–1,000 ng/ml) (Figure 6). Using this West-Eastern downstaging criteria (WE-DS) the drop-out rate in Western patients (i.e., with low frequency of living donor liver transplantation [LDLT]) was below 15% and therefore not significantly different from the UCSF-in group. In contrast, 30.4% of patients outside the WE-DS criteria experienced drop out. When comparing the WE-DS criteria with the UCSF criteria, the WE-DS group included more patients than the UCSF-in group, and only 54% of the MC-out patients would have been considered for LT according to the UCSF criteria, but 79% according to the WE-DS criteria. Nevertheless, the WE-DS group revealed the same 5-year post-LT HCC-related death rate (14.4% vs. 15%). These data confirm that the (morphometric) UCSF criteria can be easily adjusted by including biological parameters. However, even in the WE-DS-out patients, only 38% of HCC-related deaths were observed within 5 years and 42% within 10 years after LT. In other words, based on these data, 3 out of 10 WE-DS-out patients will experience drop out prior to LT and a further 3 will develop HCC recurrence after LT, but 4 out of 10 WS-DS-out patients (ITT) are theoretically good candidates for LT, but the overall number would be relatively small (i.e., 2.5% in this series) [Error! Bookmark not defined.].

On the other end of the AFP scale, it could be shown that an AFP >1,000 ng/ml is a poor prognostic factor. A large multicenter analysis has even shown that cases with pre-LT AFP >1,000 ng/ml had no survival benefit after LT (40). However, this analysis did not consider the AFP at initial referral or the treatment response. Therefore, the situation of an initial AFP >1,000 ng/ml is still unclear. It was shown in low numbers that successful downstaging is possible in patients with AFP >1,000 ng/ml, but probably achievable only in less than 20% of patients (12.5% successful downstaging in (41) and 18.8% in (19)).

**FIGURE 7:**
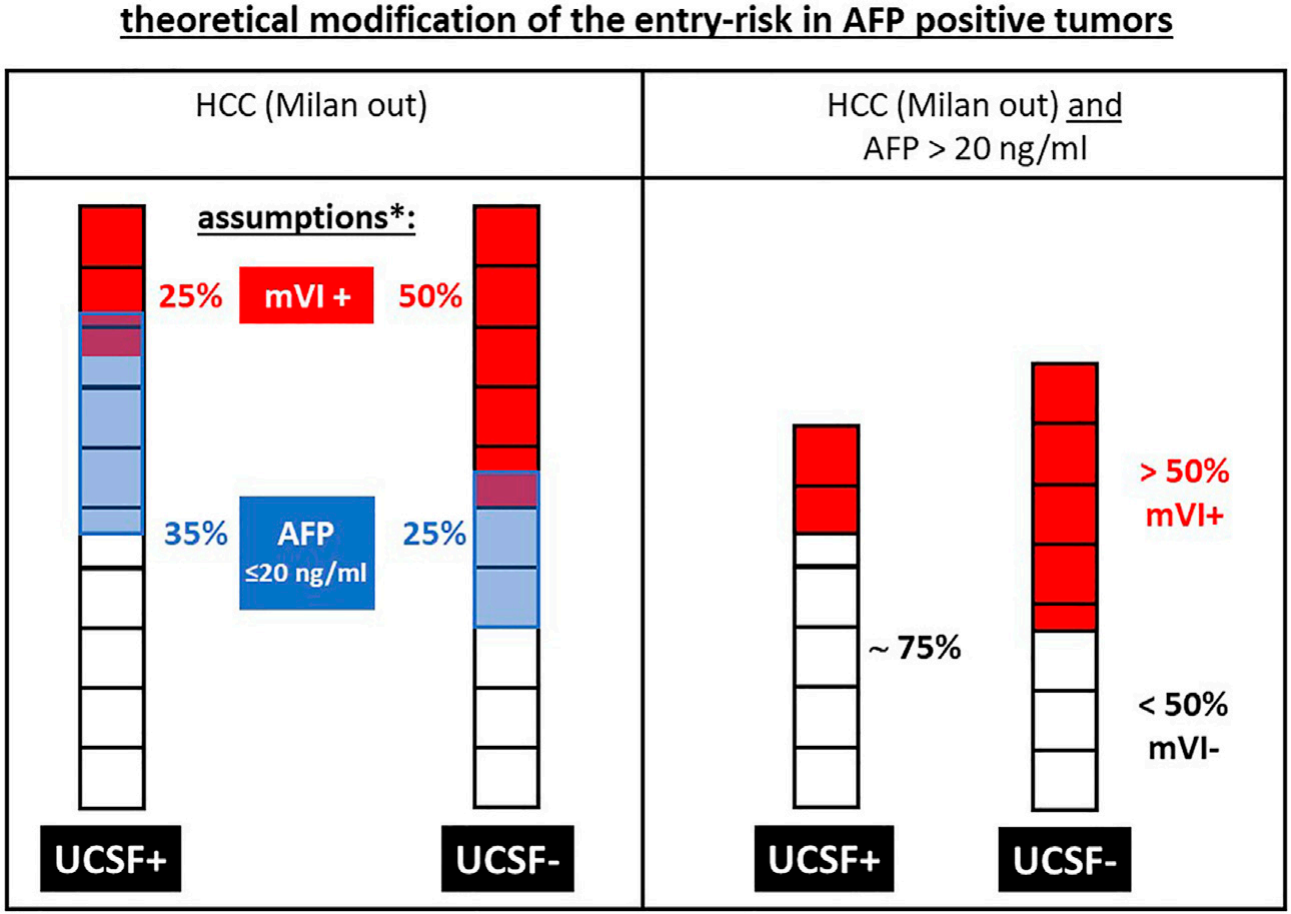
Exemplary modification of the theoretical entry risk: in AFP-positive (i.e., > 20 ng/ml) tumors the risk of microvascular invasion (mVI+) is markedly increasing in UCSF- tumors compared to UCSF+ tumors, since the remaining patients represent a “negative selection” in a group with an already increased risk of mVI+. This increasing risk by increasing AFP levels is the theoretical basis for the limitation of morphometric selection criteria with increasing AFP levels in the WE-DS criteria as well as in the adopted Metroticket 2.0 criteria as shown in Figure 6. * the assumed values for mVI+ tumors and AFP levels >20 ng/ml are based on the average data from Figure 1, but are of a theoretical nature to expertly clarify the increasing risk by patient selection criteria, which are used in addition to morphometric criteria.

In summary, the AFP level may be used as a gatekeeper prior to downstaging, as well as prior to LT. Since all proposed models are based on adjustment of probabilities, decreasing upper limits of AFP levels should be considered with increasing tumor burden to maintain the rate of futile downstaging and/or LT approaches within accepted limits (42). However, at least for patients with a tumor burden outside the WE-DS criteria, additional parameters are advisable, especially since the selection of patients with a favorable prognosis on the basis of AFP levels means the percentage of patients with predicted poor prognosis is disproportionately increasing (Figure 7). Moreover, since the WE-DS model is based solely on parameters prior to downstaging, the dynamic response to downstaging is not captured. Therefore, this model might be a useful gatekeeper. However, in patients with high tumor burden, additional parameters might be useful together with dynamic re-evaluation during downstaging protocols.

## Beyond AFP–PET, Hepatobiliary MRI, and Other Biomarkers

Whereas differentiated HCCs share a similar enzymatic activity with normal liver tissue, poorly differentiated tumors reveal a low glucose-6 phosphatase activity and high uptake of 18F-FDG. Therefore, it has been postulated that poorly differentiated tumors can be identified by means of PET positivity (PET+) (43). Therefore, PET holds the potential of being a non-invasive alternative to pre-LT tumor biopsy. Kornberg et al. showed that PET+ was the only independent predictor of tumor recurrence in patients outside the up-to-seven criteria (HR of 19.25). Independently of tumor size and number, the 5-year survival in PET-patients was 88.7% compared to 46.3% in PET+ patients (*p* < 0.001). Moreover, in the PET-patients, the percentage of mVI and G3 tumor was 12% and 9.3%, respectively, compared to 82.9% mVI and 34.1% G3 tumors in PET+ patients (*p* < 0.001). Thus, 14 of 21 poorly differentiated HCCs were PET positive (44). PET+ correlates with tumor burden, as 26% of MC-in patients were found to be PET+ compared to 47% in MC-out patients and inside the up-to-seven criteria and 48% in patients outside the up-to-seven criteria. Overall, these data confirm that morphometric criteria alone might not be ideal discriminators in MC-out patients. In a recent review, the accuracy of 18F-FDG-PET/CT for predicting mVI was 68%–88% and 55%–71% for poor tumor differentiation (43).

Data on the predictive potential of the combination of PET with AFP values are mainly derived from Asian cohorts, mainly using LDLT and often no consequent downstaging protocols. A multicentric experience from 16 Japanese LT centers showed that in multivariate analysis, exceeding MC, AFP ≥115 ng/ml, and PET+ status were independent risk factors for HCC recurrence (45). Of the 49 MC-out patients, 47% had PET+ scans and the 2-year recurrence rate in PET+ was significantly higher than in PET-patients (80.0% vs. 29.4%). A Korean analysis confirmed that PET in combination with the AFP value might be a better predictor of survival than each parameter alone. By using an AFP cut-off of 200 ng/ml they were able to define groups with a low (AFP <200 ng/ml and PET-), intermediate (PET+ or AFP >200 ng/ml), and high risk (PET+ and AFP >200 ng/ml) for tumor recurrence, leading to 5-year disease-free survival rates of 86.1%, 79.0%, and 18.5%, respectively (46).

Preliminary data on MRI criteria in transplanted patients show that the presence of satellite nodules and peri-tumoral hypo-intensity is associated with a 3-year tumor recurrence rate of 75.5% compared to 28.6% in cases of their absence (*p* < 0.001) in 32 MC-out patients (13). Imaging features of HBI MRI predicting mVI have been investigated extensively, i.e., peri-tumoral arterial enhancement, irregular tumor margin, and peri-tumoral hypo-intensity on hepatobiliary phase. All parameters correlate well with the presence of mVI and therefore warrant further investigation for transplant candidate selection (47,48). Moreover, MRI might also be helpful in identifying macrotrabecular-massive HCC with high specificity (49). Hepatobiliary MRI adding criteria beyond wash-in and wash-out has proven higher sensitivity with comparable specificity for HCC depiction in cirrhotic patients (50). Diffusion weighted imaging is another promising MRI feature to predict HCC treatment outcome. A single center trial reported lower apparent diffusion coefficient values, which predicted early recurrence after LT (51). However, the heterogenous nature of HCC, especially in larger lesions, could potentially limit the value of the diffusion technique in more advanced patients outside MC. Other biomarkers like C-reactive protein, PIVKA-II (=DCP), and the neutrophil-lymphocyte ratio have been studied in LT candidates either alone or in combination with morphometric parameters. Relevant data are only available for DCP, which has been systemically evaluated prior to LDLT in Asian centers. In this context, a DCP cut-off between 300 and 450 mAU/ml was shown to indicate a five-fold increased risk for HCC recurrence after LT (52). Its value exceeding the use of AFP and the validation in Western series is currently lacking.

Besides the AFP value, PET+ seems to be at present the only non-invasive parameter with enough clinical evidence for inclusion in clinical pathways. In contrast to MC-in patients, where even PET+ patients seem to have a good prognosis, in MC-out patients the recurrence rate is considerably increased. Therefore, a PET scan might be an additional tool for patient selection in MC-out patients, potentially in combination with other markers, like the AFP value (for dynamic AFP response during downstaging see below).

## Biology is King: Value of Dynamic Parameters During Downstaging

Prediction models, which are based on parameters available at first referral, allow only a gross a priori estimation of the HCC recurrence risk after LT. Additional dynamic parameters, such as the response to downstaging or a test of time without downstaging measures are not captured in such models. However, the response to therapy represents essential information for appropriate patient selection to further improve the predictive power, especially in MC-out patients.

Therefore, one possible approach to improve prediction could be that all potential LT candidates with HCC undergo upfront downstaging therapy irrespective of size and number. The final decision for or against LT would then be based on the treatment response (53). However, the chance of successful downstaging in (unselected) patients with a TTV >200 cm^3^ is below 5% (1 out of 22) according to data by Murali et al. (19). Whether the reported low likelihood is acceptable remains to be defined by each center, otherwise some entry criteria for the downstaging approach should be considered as discussed above. Using the TTV threshold of 200 cm^3^, the study showed that downstaging was successful in 76% (52 out of 68) of patients outside the MC. But the maximum TTV of the UCSF criteria of 144 cm^3^ indicates that the UCSF criteria are too unspecific (19). However, the TTV at initial presentation is ultimately only a random snapshot of tumor biology, reflecting a certain risk of aggressiveness, but not the individual risk. While poorly differentiated tumors (G3) outside MC might be excluded a priori by means of biopsy and/or PET scan, dynamic parameters (54) might add additional information in the remaining population, which still includes a mixture of low, intermediate, and high-risk patients. The combination of dynamic morphological and biological parameters represents not only the most concise approach of prognostic prediction prior to downstaging but is also important for patient selection during downstaging. Besides the dynamic radiological criteria (e.g., mRECIST), dynamic changes of AFP levels during the waiting time and/or downstaging procedures need be considered as dynamic changes of AFP levels are shown to be more relevant than static AFP values at initial referral [33]. Unquestionably, the progressive increase in AFP values (e.g., AFP slope >15 ng/ml (33,40)) during waiting time is associated with poor survival benefit after LT. On the other hand, a large SRTR study demonstrated that patients with AFP levels >400 ng/ml at time of listing who experienced an AFP decrease to <400 ng/ml during downstaging had a significantly improved 3-year ITT survival (81% vs. 48%) compared to those without AFP reduction (55). A simplified score has been calculated from three US centers based on tumor size and number plus AFP response on a gradual basis (200, 400, and 1,000 ng/ml). This NYCA score has been shown to provide an appropriate risk stratification [(56)].

Overall, radiological tumor progression and/or AFP progression during downstaging are associated with a significantly higher recurrence rate after LT in patients inside (57), as well as outside MC. According to available data, AFP level progression or radiologic progressive disease should therefore be considered as contraindication for LT, particularly in MC-out patients. The same applies to AFP levels >1,000 ng/ml. For AFP levels below 1,000 ng/ml, the overall risk is determined by the tumor burden. It is essential to restrict the maximum eligible tumor burden in increasing AFP categories to keep the risk of HCC recurrence within acceptable limits. This has been proven for the clinical scenarios of first patient referral, as well as for patients after downstaging and prior to LT (39) (Figure 6).

Along with AFP response, direct histological tumor response is also a known predictive factor, especially cPR after downstaging therapy as it is associated with a very low recurrence rate of 5.8% at 5 years irrespective of the initial tumor size (63). In these patients finally undergoing transplantation (i.e., in pre-selected patients), the percentage of cPR might be irrespective of the tumor burden. Mehta et al. reported cPR in 19% of patients inside MC, in 12% of UCSF-in patients, and in 19% of UCSF-out patients (58). Although these data were not collected on an ITT basis, the study underlines that tumor biology is only partially reflected by the MC. Because cPR is only definitively known after LT, it represents a difficult parameter for decision-making prior to LT. This is especially true since the radiological finding of “no vital tumor” was confirmed as cPR only in 46.5% of patients on explant pathology in a recent (2) and previous analyses (59–62).

However, advances in MRI technology might help to increase the prediction of cPR in the future [63]. Nevertheless, the radiological response to downstaging, defined by the mRECIST criteria, is a good predictor of the risk of recurrence after LT. This is underlined by the fact that radiological response to downstaging therapy was an essential parameter in order to refine the Metroticket 2.0 criteria (2). The combination of dynamic changes of tumor response to downstaging by radiological mRECIST classification and AFP levels might be able to better refine patient selection before and during downstaging procedures, especially since progressive disease during downstaging is a worse prognostic factor in patients outside MC. Furthermore, a recent analysis using a competing risk approach confirmed that non-response to neo-adjuvant therapies assessed by mRECIST increases the HCC recurrence rate after LT from <10% to >25% (64). Accordingly, the Metroticket 2.0 criteria have been modified for patients with progressive disease during downstaging, where tumor criteria have been reduced by 1–2 cm in all AFP categories (Figure 6) (2). Looking at patients who are still outside MC after downstaging (i.e., outside the “up to 6” criteria: 5 + 1 or 3 + 3), no patient with progressive disease should undergo LT according to this calculation. MC-out patients with partial response or stable disease can undergo LT when tumor burden is within the up-to-seven criteria and serum AFP is <100 ng/ml (2).

**FIGURE 8:**
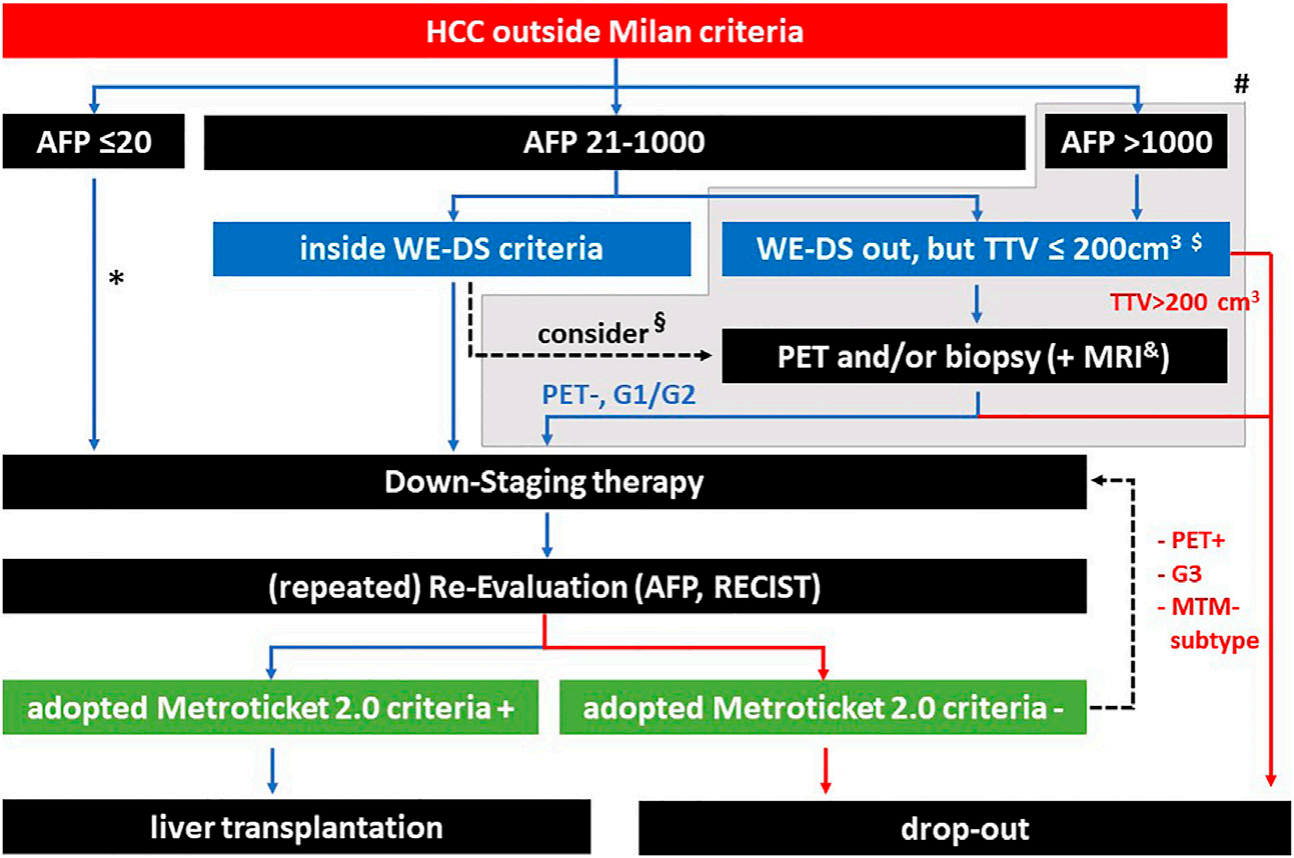
Summary of potential selection criteria before and during downstaging. * according to the WE-DS criteria, the upper limit of sum and number would be 12, above this value a PET/biopsy might be considered. § depending on the tumor burden, also inside the WE-DS criteria, additional information concerning tumor biology might be useful. # patients outside the WE-DS criteria (grey background) have a significantly elevated risk of drop out during waiting time, however, this does not preclude selection of a low percentage of patients with good prognosis after LT (depending on individual center policies and organ availability). $ with a TTV <200 cm^3^, the chance of successful downstaging is below 5% (Murali et al. [12]). & MRI techniques are evolving for the prediction of mVI and the macrotrabecular-massive (MTM) HCC subtype. Hepatobiliary MRI has proven higher sensitivity with comparable specificity for HCC depiction in cirrhotic patients.

In summary, an available single marker of measuring the risk of tumor recurrence is still far away. Tumor biology has to be assessed by tumor burden, AFP levels, and response to therapy, including the use of PET and/or tumor biopsy in selected cases. However, the selection process might be refined in the future due to expanding knowledge of available prediction parameters. An overview of potential selection parameters for and during downstaging, as discussed above, is illustrated in Figure 8. These criteria might represent only rough approximations due to differences in underlying populations and available data, as well as differences in acceptable 5-year outcome parameters in various publications. Therefore, the refined Metroticket 2.0 criteria might currently reflect the most sophisticated endpoint of downstaging, whereas the entry criteria might be applied, as depicted in Figures 6, 8.

## Future Directions

Although selection criteria for LT in HCC patients are becoming more and more sophisticated, all established parameters are still “imperfect.” In any group with a negative prognosis determined by the available criteria, there remains a small proportion of patients who will achieve long-term survival “against all odds.” This has been shown for G3 tumors, PET-positive tumors, an AFP >1,000 ng/ml, and even for patients with macrovascular invasion (65). Until more specific biomarkers are available, in subgroups with predicted poor prognosis, only exceptional cases will achieve long-term survival. Therefore, LT might be considered later on after good response to initial locoregional therapy. However, in most centers, LT will not be considered ab initio in the high-risk groups, due to the very low rate of successful downstaging and LT.

Further approaches should explore personalized prediction and therapy approaches and implement molecular knowledge in clinical practice for patients with HCC listed for LT. For this, prospective evaluation is required and intra- and inter-tumor heterogeneity and the reproducibility of molecular analysis from tumor biopsy material must be taken into account. Newer selection parameters, which are currently under investigation include newer imaging methods, like fluorocholine PET (66), molecular markers derived from biopsy material, and increasing use of liquid biopsies (67). Future data on these parameters will hopefully support a more specific risk prediction in candidates for LT outside the conventional selection criteria.

## Abbreviations

AFP, alpha fetoprotein; cPR, complete pathologic response; DS, downstaging; HCC, hepatocellular carcinoma; ITT, intention to treat analysis; LT, liver transplantation; MC, Milan criteria; MC-in, inside the Milan criteria; MC-out, outside the Milan criteria; mVI, microvascular invasion; PET, positron emission tomography; PR, partial response; SD, stable disease; TTV, total tumor volume; UCSF, University of California, San Francisco; UCSF-in, inside the UCSF criteria; UCSF-out, outside the UCSF criteria; WE-DS, West-Eastern downstaging criteria.
