# Supplementary material for: Patient Selection for Downstaging of Hepatocellular Carcinoma Prior to Liver Transplantation—Adjusting the Odds?
Source: Transpl Int. 2022 Apr 21;35:10333. doi: 10.3389/ti.2022.10333 (PMC9069348; doi:10.3389/ti.2022.10333)
Supplement: Supplementary file 1 [file DataSheet1.docx]

**Supplementary Table 1:** Published series of liver transplantation or liver resection with data on microvascular invasion in different HCC sub-categories based on tumor size, AFP and/or tumor grading (mVI: microvascular invasion, VI: vascular invasion, i.e. microvascular plus macrovascular invasion)

|  | | | **maximum tumor diameter** | | | | |
| --- | --- | --- | --- | --- | --- | --- | --- |
| **author** | **year** | **single/**  **multiple** | **<2 cm** | **2-4 cm** | **4-5 cm** | **5-8 cm** | **> 8 cm** |
| **Lauwers** | **2002** | **both** | 38% (<5cm) | | | 60% (>5cm) | |
| **Esnaola** | **2002** |  | 25% | 31% | 50% (>4cm) | |  |
| **Pawlik** | **2005** | **both** | 25% (≤3cm) | 40% (3-5cm) | | 55% (5-6.5cm) | 63% (>6.5cm) |
| **Löhe** | **2007** | **both** | 5% (<3cm) | 20% (3-5cm) | | 48% | 60% |
| **Bhattacharjya** | **2007** | **both** | 6.3% (1-2cm) | 25% (3-4cm) | 38.5% (>4cm) | |  |
| **Kim** | **2008** | **both** | 35% | 37% (2-5cm) | | 57% (<5cm) | |
| **Roayaie** | **2013** | **single** | 27% (≤2cm) |  |  |  |  |
| **Sumie** | **2014** | **both** | 42.8 (<3cm) | | 67.7% (>3 cm) | | |
| **Hwang** | **2015** | **single** | 4.1% | 13.1% | 20.6% (4-6cm) | 31.5% (6-8cm) | 30.7% (8-10cm) |
|  | | | **grading** | | | | |
| **author** | **year** |  | **G1** | | **G2** | **G3** | |
| **Esnaola** | **2002** |  | 12% | | 29% | 50% | |
| **Pawlik** | **2005** | **>5 cm** | 27% (>5cm) | | 62% (>5cm) | 61% (>5cm) | |
| **Kim** | **2008** |  | 4.2% | | 31.6 | 63% | |
| **Sumie** | **2014** |  | 0% | | 48% | 95% | |
|  | | | **AFP [ng/mL]** | | | | |
| **author** | **year** | **type of VI** | **< 20** | **20 - 400** | **400 - 1000** | **> 1000** |  |
| **Pawlik** | **2005** | mVI | 43% (<1000) | | | 64% |  |
| **Fujiki** | **2009** | mVI | 24% (<200 ng/mL) | | 56% (200-800) | 67% (>800) |  |
| **Douvoux** | **2012** | mVI | 20.3% (<100 ng/mL) | | 30.8% (100-1000) | 38.5% |  |
|  |  | VI | 24.9% (<100 ng/mL) | | 40.7% (100-1000) | 53.9% |  |
| **Liu** | **2013** |  | 18% | 23,5% |  | 32.4% | (81% HBV+) |
| **Sumie** | **2014** | mVI | 45.3% (≤100 ng/mL) | | 63.8% (>100) | |  |
| **Agopian** | **2017** | mVI | 20% | 31% (>20) | | |  |

**Supplementary Table 2:** Overview: Analyses of liver transplantation for HCC outside the Milan Criteria (MC) using downstaging approaches (# overall drop out rate on the waiting list in studies with indication of ITT-data, ^$^no DS therapy initiated before liver transplantation, n.d.: no data). Studies with mainly or exclusively living donor LT were not included due to

|  |  |  | **down-**  **staging** |  | **drop-out^#^** | **recurrence rate** | | **recurrence free**  **survival** | | **overall survival** | |
| --- | --- | --- | --- | --- | --- | --- | --- | --- | --- | --- | --- |
| **author** | **year** | **subgroup** | **to MC** | **n=** | **(%)** | **3yr.** | **5 yr.** | **3 yr.** | **3 yr.** | **3yr.** | **5yr.** |
| Yao | 2015 | MC-in | -- | 488 | -- | -- | 12 | -- | -- | 87 | 81 |
|  |  | MC-out, UCSF-in | yes | 118 | 34.8 | -- | 9.2 | -- | -- | 87 | 77.8 |
| Sapisochin | 2016 | MC-in | -- | 363 | 13.8 | -- | 13 | -- | -- | -- | 75 |
|  |  | extended Toronto | -- | 242 | 21.1 | -- | 30 | -- | -- | -- | 68 |
| Murali | 2016 | MC-in | -- | n.d. | -- | -- | -- | -- | -- | -- | 74.5 |
|  |  | MC-out | yes | 90 | 64 | -- | -- | -- | -- | -- | 79.4 |
| Chapman | 2017 | MC-in | -- | 239 | -- | 7.2 | 10.9 | 80.9 | 70.7 | 83 | 74.1 |
|  |  | MC-out, UCSF-in | yes | 35 | 48.6 | 7.7 | 7.7 | 87.2 | 87.2 | 94.4 | 85.8 |
|  |  | UCSF-out | yes | 175 | 74.3 | 6.9 | 12.1 | 73.5 | 62.8 | 73.2 | 66.2 |
| Ravaioli | 2018 | MC-in | -- | 145 | no ITT | 7 | 7.6 | -- | -- | 80 | 75 |
|  |  | MC-out, UCSF-in | yes | 43 | no ITT | 15 | 20.9 | -- | -- | 80 | 63 |
|  |  | MC-out, UCSF-in | no | 20 | no ITT | 31.6 | 31.6 | -- | -- | 68 | 62 |
|  |  | MC-out, UCSF-in | no DS^$^ | 23 | no ITT | 30 | 30.4 | -- | -- | 64 | 63 |
| Mehta | 2018 | MC-out, UCSF-in | yes | 187 | 36.4 |  | 12.7 | -- | -- | 90 | 79.7 |
| Grat | 2018 | MC-in | -- | 170 | no ITT | -- | 5.3 | 93 | 87.1 | -- | -- |
|  |  | MC-out | -- | 112 | no ITT | -- | -- | 71 | 57.9 | -- | -- |
| Affonso | 2019 | MC-in | -- | 136 | -- | -- | -- | 76,5 | 74,8 | 76,5 | 72,3 |
|  |  | MC-out | yes | 64 | 67.2 | -- | -- | 82,8 | 62,1 | 88,2 | 73,5 |
| Sinha | 2019 | MC-out, UCSF-in | yes | 133 | 35.3 | -- | 14 | 88 | 85 | 80 | 79 |
|  |  | UCSF-out | yes | 74 | 83.8 | -- | 60 | 40 | 40 | 50 | 50 |
| Toso | 2019 | MC-in | -- | 177 | no ITT | -- | 1.7 | -- | 86 | -- | -- |
|  |  | MC-out | yes | 80 | no ITT | -- | 11 | -- | 76 | -- | -- |
| Mehta | 2019 | MC-in | -- | 3276 | no ITT | 6.9 | -- | -- | -- | 83.2 | -- |
|  |  | MC-out, UCSF-in | yes | 422 | no ITT | 12.8 | -- | -- | -- | 79.1 | -- |
|  |  | UCSF-out | yes | 121 | no ITT | 16.7 | -- | -- | -- | 71.4 | -- |
| Kardashian | 2020 | MC-in | -- | 3570 | no ITT | 8.8 | 11.1 | 77 | 68.2 | 80 | 71.3 |
|  |  | MC-out | yes | 465 | no ITT | 15.1 | 19 | 64 | 59.9 | 76 | 64.3 |
|  |  | MC-out | no | 242 | no ITT | 30.4 | 34 | 62 | 53.8 | 73 | 60.2 |
|  |  | MC-out | no DS^$^ | 82 | no ITT | 18.7 | 26.1 | 67 | 56 | 73 | 61% |
| Pinero | 2021 | MC-in | -- | 912 | 4.0 | -- | 13.1 | -- | -- | -- | -- |
|  |  | MC-out, UCSF-in | yes | 83 | 69.9 | -- | 28.4 | -- | -- | -- | -- |
|  |  | UCSF-out | yes | 47 | 83.0 | -- | 32.9 | -- | -- | -- | -- |
| Degroote | 2021 | MC-in | -- | 735 | 7.5 | 10.0 | 13 | -- | -- | -- | 64.9 (4yr) |
|  |  | MC-out, UCSF-in | yes | 93 | 54.8 | 18.2 | 18.2 | -- | -- | -- | 63.0 (4yr) |
|  |  | UCSF-out | yes | 55 | 61.8 | 49.0 | 54.7 | -- | -- | -- | 47.8 (4yr) |
